# Investigation of Osteopontin (OPN) Expression in Dromedary Camel (*Camelus dromedarius*) in the First Trimester of Pregnancy

**DOI:** 10.3390/vetsci13020158

**Published:** 2026-02-06

**Authors:** Faten A. Alrashaid, Mohammed S. Moqbel, Marwa A. M. Babiker, Saeed A. Al-Ramadan

**Affiliations:** 1 Department of Anatomy, College of Veterinary Medicine, King Faisal University, P.O. Box 400, Al-Ahsa 31982, Saudi Arabia; mbabakr@kfu.edu.sa (M.A.M.B.); salramadan@kfu.edu.sa (S.A.A.-R.); 2 Livestock Sector, Almobdioon Center for Studies and Consultancy, King Abdulaziz University, P.O. Box 80088, Jeddah 21589, Saudi Arabia; moqbelmohammed@gmail.com

**Keywords:** dromedary camel, expression, histology, osteopontin, pregnancy

## Abstract

Osteopontin (OPN) is a phosphorylated acidic glycoprotein that serves various purposes across various systems. Principally, it aids in adhesion and signaling. It is an important protein related to reproduction, which has been discovered in several species, among which is the dromedary camel, where it has been detected in the endometrium and conceptuses at the time of implantation. The objective of this research is to investigate and examine the expression patterns of OPN mRNA and protein in both endometrial uterine horns with their associated fetal membranes during the first trimester of camel pregnancy. Endometrial and fetal membranes were collected from eight pregnant female camels during the first trimester of pregnancy (Days 70–110 of pregnancy). The temporospatial expression of OPN was studied in endometrial tissues and their conceptuses from the left uterine horn (LUH) and right uterine horn (RUH) using quantitative real-time PCR (qRT-PCR) and immunohistochemistry (IHC) techniques. The results showed a gradual increase in OPN mRNA expression with advancing pregnancy in both LUH and RUH of endometrial and fetal membranes tissue. OPN protein was predominantly found in the uterine luminal epithelium (LE) and fetal trophectoderm (Tr), stroma, and uterine glands. In conclusion, our findings demonstrate that OPN is consistently present in both the endometrium and fetal membranes throughout the first trimester of pregnancy. This observation advances previous knowledge by confirming OPN expression even during the early first trimester, suggesting its crucial role in supporting embryo attachment, implantation, and placentation processes in the dromedary camel.

## Simple Summary

The dromedary camel, which is economically and socially important, faces reproductive challenges such as early embryonic loss and a low reproductive rate. This study investigated the role of Osteopontin (OPN), a crucial protein involved in reproduction across many species, in the first trimester of camel pregnancy. Endometrial and fetal membranes issue samples were collected from the uteruses and conceptuses of eight pregnant camels (Days 70–110 of pregnancy) to examine the expression of OPN by using quantitative real-time PCR (qRT-PCR) and immunohistochemistry (IHC). The results showed a gradual increase in OPN mRNA expression with advancing pregnancy in both endometrial and fetal membranes tissues. OPN protein was predominantly found in the uterine luminal epithelium (LE) and fetal trophectoderm (Tr), stroma, and uterine glands. These findings suggest that OPN plays a key role in the successful reproduction of the dromedary camel by aiding in processes like embryo attachment, implantation, and the development of the placenta. Understanding this mechanism can help improve breeding success and overcome reproductive obstacles in this valuable species.

## 1. Introduction

The dromedary camel is highly adapted to waterless environments. The camel is significant socially and economically in many regions, particularly in Saudi Arabia [1,2]. However, there are many obstacles to growing the camel population, such as the inability to identify estrus and predict ovulation, which is frequently brought on by “silent heat” and seasonal breeding, and is impacted by environmental factors, including photoperiod and nutrition availability [3]. Additionally, the duration of gestation, around 13 months, and low reproductive rate cause more difficulties for the breeders [4,5]. The dromedary camel has its unique set of issues during pregnancy. Above all, the early embryonic loss, which represents a serious issue, is frequently linked to stress, hormonal imbalances, and nutritional deficits [2,4].

The camel placenta is classified as diffuse epitheliochorial, and it exhibits regional variations in villous development [3,6,7]. These fetal membranes (placenta) appose to the uterine endometrium to provide an interface necessary for metabolic exchange between the mother and the conceptus [8]. However, the process of placental development is multi stages events. In the camel, three early stages have been described: precontact, apposition, and adhesion [6]. Based on the previously described model of implantation and subsequent placentation, there must be a receptor-ligand interaction between the fetal trophectoderm and maternal luminal epithelium [9]. Among these, the ligand is the OPN that has been studied in various farm animals [10,11,12].

Osteopontin (OPN) is a multifunctional phosphorylated glycoprotein. It plays a vital role in many physiological processes. In pregnancy, OPN is implicated in implantation, decidualization, and trophoblast invasion, contributing to the establishment and maintenance of the maternal–fetal interface. It is also involved in immune modulation and tissue remodeling in many species. In reproduction, OPN function correlates with their placental types and hormonal levels. While studies have examined endometrial gene expression related to pregnancy in camels, including differences between uterine horns [13] Osteopontin has been identified as a key factor in dromedary camels [14]. The authors found that OPN levels are highest in the left uterine horn around day 12 of pregnancy during peri-implantation [15].

In camels, the role of OPN in pregnancy is a topic of ongoing research, but its potential involvement in placental development and immunomodulation is evident. This study’s primary goal is to learn more about OPNs function in implantation and examine the designs of its mRNA and protein expression in both uterine horns throughout the first trimester of camel pregnancy.

## 2. Materials and Methods

### 2.1. Animal

In this study, eight pregnant dromedary female camels (*Camelus dromedarius*) (age 7–15 years) were examined. The females were divided into two groups according to gestational age: Group 1 (G1), with a gestational age of 70–90 days (n = 4), and Group 2 (G2), with a gestational age of 100–110 days (n = 4). The fetus’s gestational age estimation was performed after slaughtering by the crown-vertebral rump length (CVRL) method according to Elwishy’s formula [16].

### 2.2. Ethics Statement

All animal procedures were approved by the Ethics Committee for Scientific Research (REC) of the Deanship of Scientific Research at King Faisal University (KFU-REC-2024-JUN-ETHICS1849).

### 2.3. Endometrial and Fetal Specimens Collection

Immediately after slaughter, the gravid uteruses were separated from the carcasses and grossly examined for any pathological changes. Endometrium with attached fetal membranes tissues were collected from both left and right uterine horns (LUH and RUH) and fixed in 10% buffered formaldehyde [17] for H&E routine stain and immunohistochemistry analysis. For qRT-PCR analysis, the fetal membranes were detached from the endometrium, specimens were collected and preserved in RNAlater^®^ solution (Ambion, Austin, TX, USA) at 4 °C for 24 h, and then stored at −80 °C until analysis.

### 2.4. RNA Extraction and cDNA Synthesis

The frozen endometrial and fetal membranes tissues were dissected to remove the myometrium layer. After homogenization, total RNA was extracted using TRIzol reagent (Bio-Rad, Hercules, CA, USA), followed by DNA purification treatment using DNase-I kit (Ambion, Austin, TX, USA). Total RNA concentration and purity were assessed using a NanoDrop spectrophotometer (260/280 and 260/230 ratios), and RNA integrity was confirmed by agarose gel electrophoresis. For cDNA synthesis, 1 µg of total RNA was reverse transcribed in a final reaction volume of 20 µL using the SensiFAST cDNA Synthesis Kit (Meridian Bioscience, Cincinnati, OH, USA) according to the manufacturer’s protocol. The thermal profile consisted of 25 °C for 10 min, 42 °C for 15 min, and 85 °C for 5 min. The resulting cDNA was diluted 1:5 and stored at −20 °C until qRT-PCR analysis [13,18].

### 2.5. Quantitative Real-Time PCR (qRT-PCR) Technique

The qRT-PCR was performed using the SensiFAST SYBR No-ROX Kit (Meridian Bioscience) on a Bio-Rad real-time PCR system. Each 20 µL reaction contained 2 µL diluted cDNA, 10 µL SYBR Master Mix, 0.4 µM forward and 0.4 µM reverse primers, and nuclease-free water. Primers for OPN and the reference gene GAPDH are used as previously designed on the NCBI Primer BLAST tool based on *Camelus dromedarius* sequences and are shown in Table 1. Thermal cycling conditions were conducted at 95 °C for 2 min for polymerase activation, followed by 40 cycles of 95 °C for 5 s, 65 °C for 10 s, and 72 °C for 20 s. All reactions were run in triplicate, including no-template and no-reverse-transcription controls. Gene expression was quantified using the 2^−ΔΔ*C*T^ method and normalized to GAPDH.

### 2.6. Hematoxylin and Eosin Techniques

For H&E staining, the tissues were placed in 10% neutral buffered formalin solution immediately after slaughter and incubated for 72 h. After fixation, the collected tissues were then processed by the routine paraffin embedment technique, Briefly, specimens were dehydrated in graded concentrations of ethanol before they were cleared in xylene and embedded in paraffin. Serial sections of 4–5 µm thickness were cut and carefully collected on slides for staining with H&E. The tissue sections were deparaffinized and rehydrated through xylene and graded alcohols to distilled water before they were stained with Ehrlich’s hematoxylin for 10 min. The sections were differentiated with two quick dips in 2% acid alcohol, then washed in the tap water for 10 min. After that, they were stained with eosin for 4 min followed by dehydration through 90% and 100% alcohols. Finally, the slides were cleared in xylene and mounted for microscopic examination.

### 2.7. Immunohistochemistry Technique

Endometrial and fetal membranes tissue sections (5 µm) were deparaffinized and rehydrated. Antigen retrieval was carried out for 10 min using sodium citrate buffer (pH 6) using a microwave oven. After cooling down, the sections were coated with endogenous peroxidase blocking buffer for 15 min, then treated with 30 min of protein blocking buffer and washed with 0.1% (*v*/*v*) Tween 20 in Tris-buffered saline (TBST: 3 × 5 min). Sections were then incubated with rabbit anti-osteopontin (Sigma Aldrich, Saint Louis, MO, USA, HPA027541, 1:100) overnight at 4 °C. After washing with TBST, a secondary biotinylated goat anti-rabbit antibody (ab64261, Abcam, Cambridge, UK) was added for 1 h at room temperature (RT). After washing, the slides were incubated with the streptavidin-HRP conjugate for 20 min. The sections were then treated with freshly made 3,30-diaminobenzidinetetra-hydrochloride chromogen substrate (DAB/chromogen solution) for 10 min to visualize the color. The sections were dehydrated, cleaned, and mounted for microscopic examination. To check the specificity of the primary antibody, the same concentration of normal rabbit immunoglobulin (rIgG) (Abcam, ab171870) was used as a negative control. Each step was followed up in compliance with the guidelines provided by the manufacturer (Abcam PLC, Waltham, MA, USA). Images were taken using 20× (NA 0.7) and 40× (NA 0.85) objective lenses on a digital microscope (Leica DM6000 B, Wetzlar, Germany) equipped with a digital camera (Leica DFC420, Germany) and LAS V4.2 software. The immunostaining intensity was quantified in triplicate using the Fiji Imagej software version 1.53q. https://imagej.net/software/fiji/ (accessed on 2 February 2025) [17].

### 2.8. Statistical Analysis

All data were loaded into the GraphPad Prism^®^ 5 software. The significance of differences between groups was assessed using the One Way ANOVA, and multiple comparisons were performed using Tukey’s post hoc test. The data were presented as means ± standard errors. A *p*-value of less than 0.05 (*p* < 0.05) was considered statistically significant.

## 3. Results

### 3.1. OPN mRNA Expression

#### 3.1.1. The Endometrium

**Figure 1:**
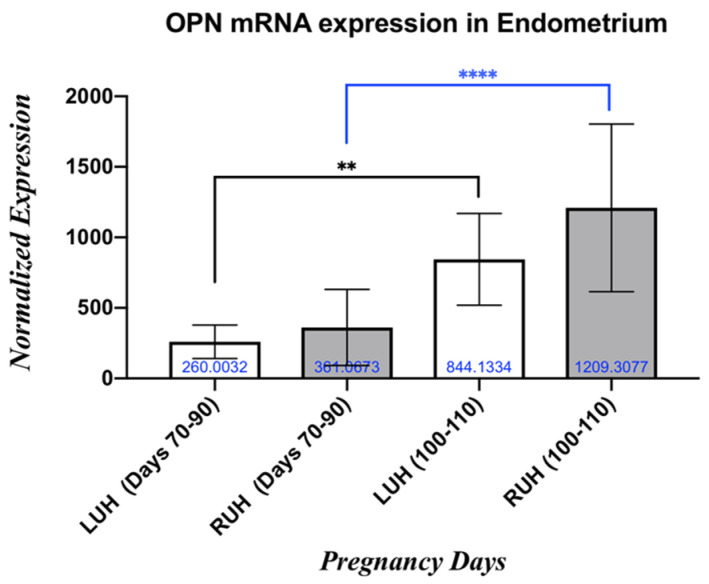
OPN mRNA expression’s fold differences in LUH and RUH of endometrial tissues during the first trimester (Days 70–90 and Days 100–110) of pregnant female camels. The black line indicates the differences in LUHs comparison, while the blue line indicates the RUHs comparison. ** is considered as *p* < 0.01, **** is considered as *p* < 0.0001.

In both LUH and RUH, the OPN mRNA expression was upregulated during the pregnancy from Days 70–90 and Days 100–110. In LUHs, OPN mRNA level was significantly (*p* < 0.05) higher, about 3.25-fold, on Days 100–110 compared to Days 70–90. Similarly, in RUHs, the OPN mRNA level was higher, about 3.35-fold, on Days 100–110 compared with the level on Days 70–90. In comparing LUH and RUH within the same group, no significant differences in OPN expression are seen in all pregnant groups (Figure 1).

#### 3.1.2. The Fetal Membrane

**Figure 2:**
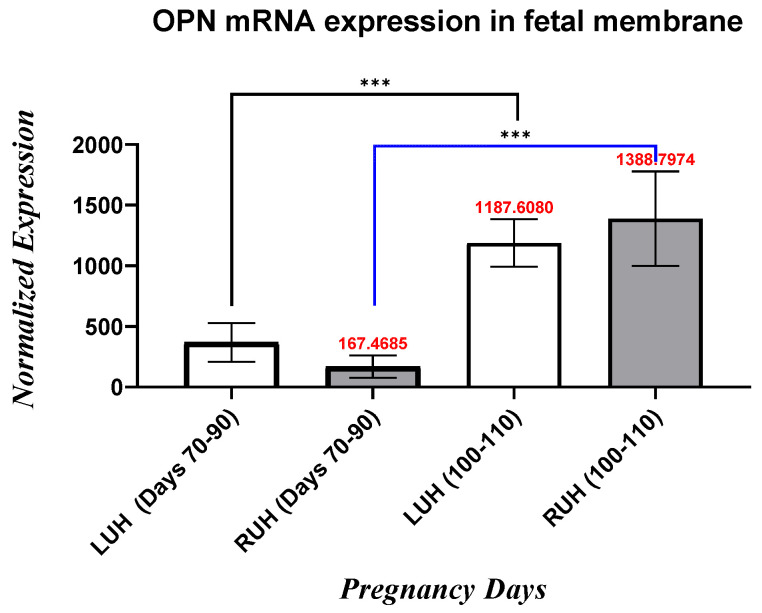
OPN mRNA expression’s fold differences in LUH and RUH of fetal membranes tissue during the first trimester (Days 70–90 and Days 100–110) of pregnant female camels. The black line indicates the differences in LUHs comparison, while the blue line indicates the RUHs comparison. The statistically significant value is considered as (*p* < 0.001), and the differences in levels are marked by ***.

In LUHs, the *fetal membranes* OPN mRNA expression was significantly (*p* < 0.05) higher (3.23-fold) in Days (100–110) compared with Days (70–90) Whereas, in the RUHs, the OPN mRNA expression level exhibited a significant (*p* < 0.05) sharp increase of (8.29-fold) in Days (100–110) compared with that in Days (70–90). When comparing LUH and RUH with the same group, no significant differences in OPN mRNA expression level are seen in all pregnant groups (Figure 2).

### 3.2. Histological Analysis

**Figure 3:**
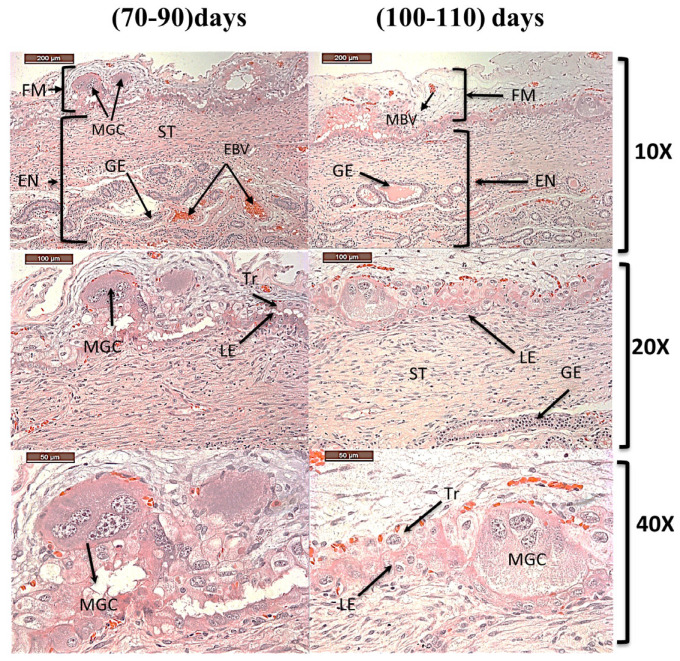
Photomicrograph in cross section of first trimester *Camelus dromedarius* in the left horn indicating fetal membrane (FM) and endometrium (EN), trophoblast (Tr), stroma (ST), fetal membranes blood vessels (MBV), luminal epithelium (LE), uterine glands (G), endothelial blood vessel (EBV), multinucleated giant cells (MGC), with different magnifications (H&E 10×, 20× and 4×).

Histological evaluation of the dromedary camel endometrium during the first trimester of pregnancy demonstrated a well-defined epitheliochorial placental interface, characterized by preservation of the maternal endometrial surface epithelium in close apposition to the fetal trophoblast, with no histological evidence of trophoblastic invasion, consistent with a diffuse microcotyledonary type of placentation (Figure 3). The maternal endometrium comprised a luminal epithelium (LE) formed by tall columnar epithelial cells overlying a well-developed lamina propria (LP) containing simple tubular uterine glands (G), which occasionally exhibited branching in the deeper endometrial regions, in addition to an extensive maternal vascular network supporting fetomaternal exchange. The fetal compartment was represented by the chorionic membrane (CM), dominated by trophoblastic cells supported by the embryonic stroma (ES) of the allantoic membrane (ALM). The chorioallantoic membrane contributed substantially to placental organization, with the chorionic epithelium adhering appositionally to the maternal epithelium to form diffuse microcotyledonary units that facilitate nutrient and gas exchange, while the allantois displayed irregular folding accompanied by marked vascularization. Multinucleate trophoblast giant cells (MGC) were observed sporadically along the trophoblastic layer throughout the examined gestational period (Figure 3).

### 3.3. Localization of OPN Protein

**Figure 4:**
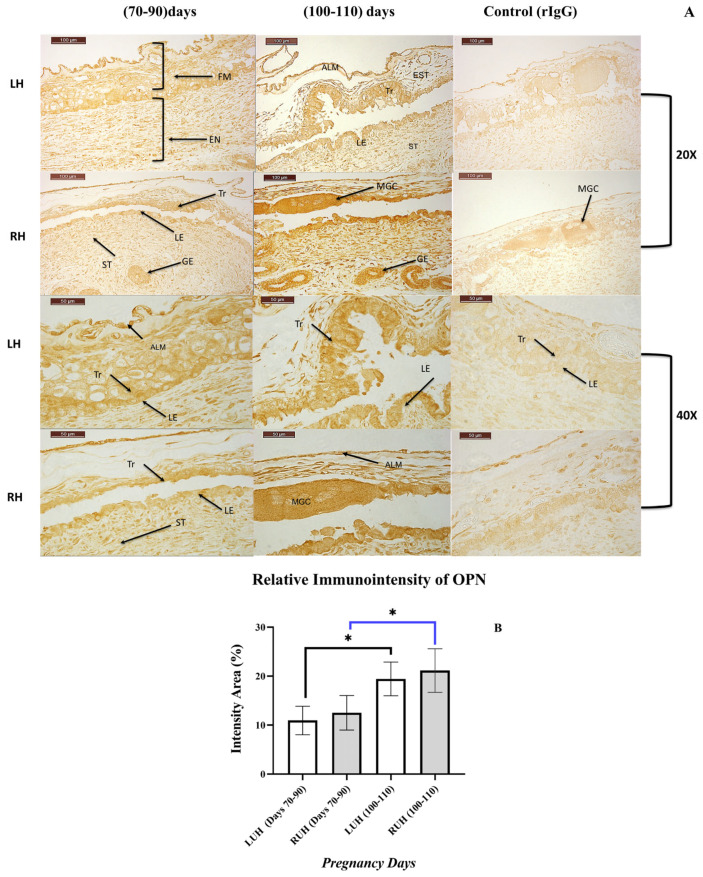
(**A**) Immunochromogenic detection and localization of OPN protein in the LUH and RUH of pregnant camels during the first trimester (Days 70–90 and Days 100–110). Positive OPN immunoreactivity was observed in both the fetal membranes (FM) and endometrium (EN), with distinct staining detected in the trophectoderm (Tr), multinucleated giant cells (MGC), allantoic membrane (ALM), fetal membranes stroma (EST), uterine luminal epithelium (LE), glandular epithelium (GE), and uterine stroma (ST). OPN immunoreactivity was localized predominantly at the apical region and within the cytoplasm of LE and Tr (arrowheads), as well as in the epithelium of uterine glands (GE) and both fetal membranes and uterine stromal compartments (EST and ST). Negative control sections are shown in the right column. Scale bars represent 100 µm for 20× magnification and 50 µm for 40× magnification. (**B**) Semi-quantitative analysis of OPN immunostaining intensity in pregnant female camels. Relative immunointensity analysis demonstrated an upregulation of OPN expression in both the LUH and RUH at days 100–110 of pregnancy. Statistical significance is indicated as *. The black line represents LUH comparisons, whereas the blue line represents RUH comparisons across all pregnant camels.

Immunohistochemical evaluation demonstrated a distinct, stage-dependent pattern of OPN protein localization in uterine tissues and fetal membranes of pregnant dromedary female camels during the first trimester (days 70–90 and days 100–110 of gestation). OPN immunoreactivity was detected in both LUH and RUH, with positive immunostaining observed in the endometrium and associated fetal membranes. Spatially, OPN immunoreactivity was distributed across the endometrial-fetal tissues and localized within the trophectoderm, multinucleated giant cells, allantoic membrane, fetal stroma, uterine luminal epithelium, glandular epithelium, and uterine stroma. At the cellular level, OPN staining was predominantly confined to the apical membrane and cytoplasmic compartments of luminal epithelial and trophectodermal cells, with additional immunoreactivity detected in glandular epithelial cells and stromal compartments of both fetal and maternal origin (Figure 4A). Semi-quantitative image analysis revealed a significant (*p* < 0.05) increase in OPN immunoreactivity at days 100–110 compared with days 70–90 of pregnancy in both uterine horns (Figure 4B).

## 4. Discussion

This study provides a foundational analysis of the temporal expression of osteopontin (OPN) during the first trimester of pregnancy in the dromedary camel (*Camelus dromedaries*). The primary finding is a consistent upregulation of OPN mRNA and protein in the uterine endometria and their corresponding fetal membranes. In the present study, the localization of OPN within the luminal and glandular epithelium, uterine stroma, and fetal membranes, particularly the trophectoderm, indicates its participation in a multi-stage cascade that supports successful pregnancy in the dromedary camel. Similar patterns of compartment-specific OPN expression have been reported in sheep, where OPN is distributed across the uteroplacental interface and contributes to conceptus adhesion and trophoblast-endometrial communication [19]. These parallels highlight a conserved functional role for OPN among mammals. By comparing our findings with established roles of OPN in other species, we emphasize the potential importance of OPN in the unique reproductive physiology of the dromedary camel and suggest directions for future research exploring its regulatory mechanisms during early gestation. The role of OPN in mediating the intricate processes of embryo implantation and placentation appears to be a conserved evolutionary strategy among placental mammals. The current study’s observations in the dromedary camel align with a well-established mechanism in species ranging from humans to ruminants. As a multifunctional phosphorylated glycoprotein, OPN is known to work as a critical “bridging ligand” that facilitates cell–cell adhesion and communication between the maternal endometrium and the invading trophoblast of the conceptus [20]. In the sheep, OPN is a component of the uterine histotroph, a nutritive secretion from the endometrial glands. Furthermore, OPN expression in the endometrial stroma increases significantly during ovine pregnancy, indicating a decidualization-like differentiation of the stromal cells in the ewe [21]. Osteopontin (OPN) is hypothesized to bind to integrin receptors expressed on the luminal epithelium [20]. Osteopontin (OPN) binds to specific integrins on cell surfaces, particularly the αvβ3 receptor, which is crucial during the implantation window in humans, pigs, and sheep [22].

Furthermore, successful placentation requires a receptive uterine environment, which is formed by the regulated invasion of trophoblast cells into the maternal tissue [20]. OPN has been shown to be a key regulator of this process. In mice, OPN is expressed in the decidua and is essential for trophoblast invasion, in part by regulating the expression and enzymatic activity of matrix metalloproteinase-9 (MMP-9) [23]. However, the decidualization is not a characteristic feature of the livestock animals and camelids [24,25]. Yet, some degree of decidualization has been reported in the uterine stroma of sheep [26] and the dromedary camel [25]. Furthermore, OPN expression in stromal cells is regulated by progesterone, and increased OPN can promote the expression of genes related to decidualization and angiogenesis [11]. Therefore, detection of OPN protein in the uterine stroma of the dromedary camel, with its staining intensity increasing with gestational age, points to a potential role in regulating stromal cell differentiation and supporting the vascular and tissue remodeling necessary for the developing placenta. The current study showed that the dromedary camel’s long gestation period of approximately 13 months necessitates a robust and well-developed placenta, and the sustained high expression of OPN throughout the first trimester suggests its central role in building this critical structure. The study’s findings; therefore, align with a conserved, multi-functional role for OPN while hinting at its species-specific adaptation to the camel’s unique reproductive strategy.

An unambiguous feature of the camel uterus is the inequality of the size of both horns, with the left one being larger than the right one [27]. Another unique and significant feature of dromedary camel reproduction is the near-exclusive occurrence of pregnancy in the left uterine horn (LUH), with a reported frequency of 98% regardless of the origin of the ovulated oocyte [27,28]. In addition, pregnancies initiated in the right uterine horn (RUH) frequently outcome in early embryonic death, a phenomenon that contributes to the low reproductive efficiency of the species [29,30,31]. This well-documented reproductive asymmetry presents a compelling context for the current study’s findings on OPN expression. However, our data showed no significant difference in OPN mRNA expression between the LUH and RUH during the later first trimester (days 70–90 and 100–110), which appears somewhat different from an earlier study that reported significantly higher OPN expression in the LUH around day 12 of pregnancy, during the peri-implantation period [15]. This is not necessarily a contradiction but rather a critical temporal observation that sheds light on the dynamic role of OPN. The initial, asymmetrical upregulation of OPN in the LUH during the peri-implantation period (around day 12) may be a pivotal prerequisite for successful embryo adhesion and the establishment of a receptive uterine environment [15]. This early, localized expression could function as a biological “gatekeeper” that dictates the success of implantation. By the time the current study’s samples were collected, between days 70 and 110 of gestation, implantation would have been long established. The non-significant differences observed later in this study, the first trimester, could thus represent a subsequent, symmetrical upregulation of OPN in both the endometrium and the conceptus of the successfully pregnant horn. The right horn, lacking a viable embryo, would not exhibit this robust, sustained upregulation. Therefore, OPN’s expression is not simply a general marker of pregnancy but a potential factor whose early, localized distribution may contribute to the observed horn dominance. In contrast, its later, more generalized expression is a consequence of an already successful implantation. In this respect, the higher OPN expression in the LUH during the critical window for embryo migration and implantation could create a more conducive environment that actively retains the migrating embryo, further contributing to horn dominance. This intricate interplay between uterine anatomy, embryo migration, and a key molecular signal, such as OPN, highlights a complex mechanism underlying a significant reproductive challenge.

Moreover, OPN has been revealed to play a crucial role in immune regulation and vascular remodeling at this interface in other species [22]. The presence of OPN in the uterine stroma of the dromedary camel, a region of extensive decidualization and immune cell presence, suggests it may modulate the activity of these cells to promote immune tolerance. The current study’s finding of OPN in the uterine stroma and its increasing intensity as pregnancy progresses points to its potential involvement in these processes. A previous study had mentioned that OPN is involved in angiogenesis and vascular remodeling [22]. Therefore, OPN in the dromedary camel may be acting as a coordinated signal that first facilitates embryo adhesion, then supports trophoblast invasion and decidualization-like, and finally contributes to the immune and vascular remodeling necessary for a successful, long-term pregnancy. This multifaceted mechanism, orchestrated by a single molecule, is essential for sustaining the camel’s long gestation. Aberrant OPN expression could; therefore, be a contributing factor to early embryonic death, a recognized problem in camel reproduction [15].

While this work sheds light on the role of OPN in dromedary camel pregnancy, it is important to recognize its limitations. The statistical power and generalizability of the findings are limited by the eight-camel sample size. The study’s focus on the later stages of the first trimester (days 70–110) provides a crucial snapshot but does not capture the critical peri-implantation period (approximately days 8–12), which previous research has shown is a time of significant OPN expression differences between uterine horns [16].

Understanding the molecular mechanisms in this highly adapted species could yield insights applicable to other mammals, including humans. Future research should build on these findings with a more comprehensive approach. A key recommendation is to conduct a longitudinal study of OPN expression from ovulation through the entire gestation period. This would be essential for fully elucidating its temporal dynamics, particularly during the peri-implantation period, and for rigorously testing the hypothesis of a temporal shift in OPN expression that contributes to left uterine horn dominance. Such a study would provide a complete picture of OPNs role at each stage of pregnancy. Additionally, a more detailed investigation into the molecular mechanisms and hormonal regulation of OPN expression is warranted. This should include identifying the specific integrin receptors that OPN binds to at the maternal–fetal interface and examining the roles of hormones like progesterone and estrogen, which are known to regulate OPN in other species [22]. Functional studies, either in vitro using camel cell lines or in vivo models, could characterize OPN’s precise role in trophoblast invasion, decidualization, and uterine vascular remodeling.

Finally, the potential for OPN to serve as a biomarker for uterine receptivity and early embryonic loss should be explored. High rates of ovulation failure and early embryonic death, particularly in aided reproductive technologies like artificial insemination and embryo transfer, are significant challenges in camel breeding programs [31]. Identifying a reliable molecular marker like OPN could aid in improving these technologies, which would have significant economic and social implications, particularly in regions where the dromedary camel is of high value.

## 5. Conclusions

The temporospatial expression of OPN mRNA and protein in the endometrium and fetal membranes of dromedary female camels was determined during the first trimester of pregnancy. The upregulation of OPN expression and its specific localization pattern, particularly at the maternal–fetal interface, implies its crucial role in the implantation process during the first trimester of pregnant female camels. The findings align with a conserved role for OPN in embryo adhesion, placentation, and stromal remodeling, as observed in other mammalian species. This research represents a significant step toward unraveling the complex molecular mechanisms governing camel pregnancy. Understanding the molecular mechanisms orchestrated by OPN, particularly its role in implantation and stromal remodeling, provides a crucial foundation for developing strategies to mitigate early embryonic loss and eventually enhance the low reproductive efficiency of this socioeconomically important species.

## Figures and Tables

**Table 1:** Primers used for the qRT-PCR.

Gene Name	Forward	Reverse	Accession Number
OPN	GTTCCGACGAGTCTCACCAT	GGAGTGAAAACTGCGGTTGC	XM_010983105.2
GAPDH	CCTGGAGAAACCTGCCAAATA	CTATTGAAGTCGCAGGAGACAA	EU331417.1

## Data Availability

The original contributions presented in this study are included in the article. Further inquiries can be directed to the corresponding author.
